# MAPK Signaling Pathway Is Essential for Female Reproductive Regulation in the Cabbage Beetle, *Colaphellus bowringi*

**DOI:** 10.3390/cells11101602

**Published:** 2022-05-10

**Authors:** Zijie Huang, Zhong Tian, Yulian Zhao, Fen Zhu, Wen Liu, Xiaoping Wang

**Affiliations:** Hubei Key Laboratory of Insect Resources Utilization and Sustainable Pest Management, College of Plant Science and Technology, Huazhong Agricultural University, Wuhan 430070, China; zijiehuang168@163.com (Z.H.); tianzhongwy@163.com (Z.T.); ylzhao188536@163.com (Y.Z.); zhufen@mail.hzau.edu.cn (F.Z.); liuwen@mail.hzau.edu.cn (W.L.)

**Keywords:** MAPK signaling pathway, reproduction, germline stem cells

## Abstract

The mitogen-activated protein kinase (MAPK) signaling pathway is a well-conserved intracellular signal transduction pathway, and has important roles in mammalian reproduction. However, it is unknown whether MAPK also regulates insect reproductive mechanisms. Therefore, we investigated the role of the MAPK signaling pathway in ovarian growth and oviposition in the cabbage beetle *Colaphellus bowringi*, an economically important pest of Cruciferous vegetables. As an initial step, 14 genes from the extracellular regulated protein kinase (ERK), c-Jun N-terminal kinase (JNK), and p38 MAPK (P38) cascades were knocked down using RNA interference (RNAi). The results revealed that RNAi knockdown of *MAPK-ERK kinase* (*MEK*), *ERK*, *Kinase suppressor of RAS 2* (*KSR2*), and *P38* induced ovarian development stagnation, low fecundity, and decreased longevity, which indicate that ERK and P38 signaling pathways are important for female *C. bowringi* survival and reproduction. The potential regulatory role of ERK and P38 pathways in the female reproductive process was investigated using quantitative real-time PCR. We found that ERK pathway possibly regulated ecdysone biosynthesis and P38 pathway possibly involved in the germline stem cell (GSC) development and differentiation. Our findings demonstrated the importance of the MAPK signaling pathway in the female reproduction of insects, and further enhanced the molecular mechanism of female reproductive regulation in insects.

## 1. Introduction

In eukaryotic organisms, the mitogen-activated protein kinase (MAPK) signaling pathway is a well-known and well-conserved intracellular signal transduction pathway. This pathway is essential for yeast to mammalian cell differentiation, proliferation, apoptosis, and metabolism [1,2,3]. Extracellular signals such as growth factors, differentiation factors and stress stimulate activators, which primarily consist of small G proteins (e.g., HRAS) that must activate at least three layers of protein kinase known as MAPK kinase kinase (MAPKKK)-MAPK kinase (MAPKK)-MAPK cascades. The intracellular upstream signal is then transmitted to the downstream response molecule with sequential phosphorylation of MAPK cascades [2,4]. The cascades can be classified as extracellular signal-regulated kinase (ERK) 1/2, c-Jun N-terminal kinase (JNK), P38 MAPK (P38), ERK3/4, and ERK7/8, with the first three being the most studied [5,6]. The activation and function of the ERK, JNK and P38 modules vary. The ERK signaling pathway is important in cell proliferation and differentiation following its stimulation by growth factors [4,7]. Growth factors and stress activate the JNK module, and it is required for cell survival and morphogenesis [8,9], while the P38 cascade is a stress-induced cascade that is responsible for apoptosis and inflammation [10,11].

According to the above explanation, ERK, JNK, and P38 pathways are involved in various physiological processes via different mechanisms. In amphibians, fish, and mammals, the importance of the MAPK signaling pathway in reproduction has been thoroughly studied, for instance, ERK cascade regulates the meiosis of oocytes [12,13], the fertilization of eggs [13], and the growth of ovarian granulosa cells [14]. The JNK pathway is responsible for follicle cell development [15,16], and P38 signaling regulates apoptosis in the ovarian cells [17]. At the cellular level, few studies have explained the main role of ERK signaling pathway in insects and *Drosophila melanogaster* [18,19]. In addition, a recent study showed that MAPK signaling pathway was involved in female reproduction of *Bactrocera dorsalis* [20]. However, there is a lack of evidence in systemic research that supports the role of the MAPK signaling pathway in regulating the female reproduction.

Several studies have linked female insect reproduction to population growth and maintenance. The ovary is a major reproductive organ in female insects, and its development is usually divided into two stages: previtellogenesis and vitellogenesis. It has been demonstrated that during the previtellogenesis process in *D. melanogaster*, germline stem cells (GSC) differentiated into various cells such as oocytes and nurse cells, demonstrating the importance of GSC development and differentiation [21,22], with *protein nanos* (*Nanos*) and *DEAD-Box Helicase Vasa* (*Vasa*) being the most commonly used marker genes [23,24]. Vitellogenesis is a process by which the vitellogenin (Vg) is synthesized primarily in fat bodies and is deposited in the ovaries. It is regarded as key event in female reproduction owing to its induction of oocyte maturation [25,26]. Previous research has indicated that juvenile hormone (JH) and 20-hydroxyecdysone (20E) are the primary regulators of female reproduction [27,28,29]. Many pathways, such as the insulin and target of rapamycin (TOR) signaling pathways, are involved in previtellogenesis and vitellogenesis [30,31]. Several studies have suggested that ERK, JNK, and P38 signaling pathways in insects may be linked to JH, 20E and insulin signaling pathways [32,33,34]. However, there is limited evidence to support the claim that these pathways regulate the female reproduction. As a result, we conducted this research to better understand how these pathways regulate reproduction in female insects.

In this study, we first identified 14 candidate genes from ERK, JNK, and P38 signaling pathways before performing RNAi knockdown on the cabbage beetle *C. bowringi*, an economically significant pest of Cruciferous vegetables. The larvae and adults of *C. bowringi* mainly fed on leaves of host vegetables, reducing the yield of vegetables and causing economic losses [35], which is due to the strong fecundity of female adults. Each female adult could produce 600–1000 eggs, had a pre-oviposition period of 3–4 days and egg-laying period of 30–60 days [36]. Most prominently, our previous investigations revealed that JH and 20E signaling might regulate *C. bowringi* ovarian development by regulating Vg synthesis [37,38,39]. This study aimed to elucidate ERK and P38 signaling pathways; the former might be involved in mediating 20E signaling for production regulation and the latter might be responsible for GSC development and differentiation. Furthermore, the current study investigated the possible involvement of the MAPK signaling pathway for female reproduction in *C. bowringi*, expanding our understanding of female reproductive regulation in insects.

## 2. Materials and Methods

### 2.1. Experimental Animals

Approximately 500 *C. bowringi* adults were captured from the natural population in Xiushui County (29° N, 114° E), Jiangxi Province, China. The beetles were raised at 25 °C and 70% relative humidity and were fed radish leaves (*Raphanus sativus* var. *longipinnatus*), according to previous study [40]. The newly hatched larvae were kept under 12 h light and 12 h dark cycles and were allowed to undergo oocyte generation, oocyte maturation, and thus spawn 3–4 days after eclosion (PE).

### 2.2. Gene Cloning and Sequence Analysis

**Figure 1:**
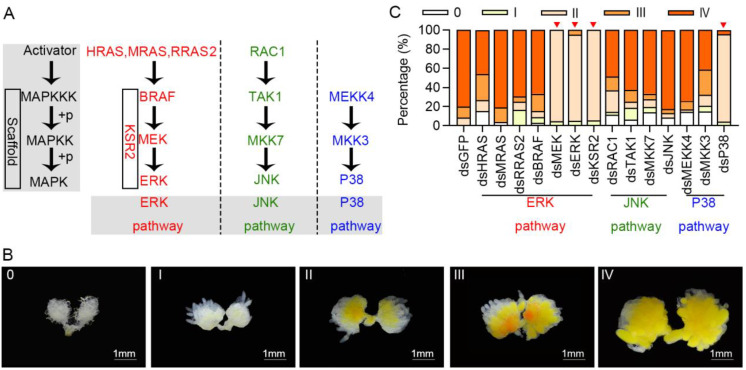
RNAi knockdown of candidate genes in the MAPK signaling pathway in female *C. bowringi.* (**A**) The pathway diagram shows 14 candidate genes from ERK, JNK, and P38 pathways in the MAPK signaling pathway. (**B**) Ovary phenotypes of *C. bowringi* at 0, I, II, III, and IV development grades. Grade 0, the ovary is white and transparent, and the ovariole presents silk thread shape; Grade I, the ovariole is obviously tubular, and the yolk deposition begins; Grade II, yolk gradually deposited and ovary gradually expanded; Grade III, a small amount of mature eggs have appeared, and there are no eggs in the lateral oviduct; Grade IV, the ovariole is full of mature eggs, and mature eggs can also be seen in the lateral oviduct. (**C**) The development grades of candidate genes knocked down at 4-day PE (*n* = 23–37). Genes marked with red triangles significantly inhibited ovarian development.

Based on a reference pathway (map04013) from the Kyoto Encyclopedia of Genes and Genomes (KEGG) database and previous research [41,42], *C. bowringi* transcriptome database (PRJNA575933) was used to retrieve the sequences of 14 candidate genes from the activator, MAPKKK, MAPKK, and MAPK members of ERK, JNK, and P38 cascades (Figure 1A and Table S1). Open reading frame (ORF) sequences of candidate genes were cloned using TA cloning and submitted to Genbank (Figure S1 showing Genbank accession numbers for these 14 genes). ExPASy translate tool (https://web.expasy.org/translate/, accessed on 20 January 2022) was used to deduce all corresponding protein sequences. MEGA 6 software created 14 rooted phylogenetic trees using the neighbor-joining method based on these sequences. The Simple Modular Architecture Research Tool (SMART) (http://smart.embl-heidelberg.de/, accessed on 21 February 2022) predicted the functional protein domains.

### 2.3. RNA Extraction and Quantitative Real Time-PCR (qRT-PCR)

Total RNA was extracted using TRIzol (Takara, Kyoto, Japan) per manufacturer specifications. The PrimeScript RT reagent Kit with gDNA Eraser (Takara, Kyoto, Japan) was used to perform cDNA reverse transcription according to manufacturer’s instructions. qRT-PCR was then conducted on an ABI QuantStudio 6 Flex (Thermo Fisher Scientific, Waltham, MA, USA) using the corresponding primers (Table S2) and TB Green Premix Ex TaqII (Takara, Kyoto, Japan). A total volume of 10 μL was used for qRT-PCR reaction, which included 5 μL TB Green Premix Ex Taq II, 2.6 μL nuclease-free water, 0.2 μL forward and reverse primers (10 μM), and 2 μL of cDNA. The qRT-PCR reaction condition was 95 °C for 30 s, followed by 40 cycles of 95 °C for 5 s, 60 °C for 30 s, 95 °C for 15 s, 60 °C for 1 min, and 95 °C for 15 s. For the normalization of gene expression, *ribosomal protein L19* (*RPL19*) and *Actin1* were used as reference genes [43]. The 2^−ΔΔCT^ method was used to assess relative expression levels [44] with three biological replicates and three technical replicates each.

### 2.4. RNAi Experiments

All double-stranded RNAs (dsRNAs) were synthesized using RNAi primers (Table S3) and a T7 transcription kit (Thermo Fisher Scientific, Waltham, MA, USA) according to manufacturer’s instructions. As previously described, RNAi experiments were used to reduce gene expression in female pupae [45]. About 2 μg dsRNAs of target genes were dissolved in 200 nL nuclease-free water and microinjected into the dorsal internode membrane of female pupae as a control, along with dsRNA of green fluorescent protein (GFP).

Tissues from 15 females were obtained at 4 days PE for each biological replicate, including heads, midguts, fat bodies, and ovaries. For total RNA isolation and qRT-PCR, each treatment had three biological replicates. At 4-day PE, ovarian development grades (*n* = 18–37) and ovarian sizes (*n* = 10–13) were recorded and measured, as instructed in previous studies [45,46]. To identify if the down-regulation of target genes affects female survival and fecundity, each newly emerged female beetle was treated with dsRNA during the pupal stage and reared in the same condition in a petri dish (diameter × height = 3 × 3 cm). Every day, fresh radish leaves were provided for feeding and oviposition. Female adult survival was recorded daily until all female adults died. The eggs laid from each female adult were recorded daily from 3–4 days PE until death, as the pre-oviposition period is 3–4 days.

### 2.5. Statistical Analysis

All experimental results were analyzed statistically using SPSS 14 (SPSS Inc., Chicago, IL, USA) and GraphPad Prism 8 (GraphPad Software Inc., San Diego, CA, USA). One-way ANOVA was used to determine the significance of tissue expression patterns, followed by Tukey’s LSD tests (α = 0.05). To determine the significance of survival rates, a Kaplan–Meier (KM) survival estimate with a log-rank test was used. For other variables, significance differences were achieved using an Independent-samples *t*-test, without significant difference, * *p* < 0.05, and ** *p* < 0.01. Means and standard deviations (SD) were used to represent values.

## 3. Results

### 3.1. RNAi Knockdown of Candidate Genes in MAPK Signaling Pathway in C. bowringi Females

Fourteen candidate genes from ERK, JNK and P38 pathways were retrieved from the transcriptome database, covered MAPK pathway members including activator, MAPKKK, MAPKK, and MAPK (Table S1). These 14 candidate genes were then cloned and sequenced, and their positions in the MAPK signaling pathway are shown in Figure 1A. A total of 14 phylogenetic trees were built using protein sequences of candidate genes. Phylogenetic analyses revealed that all candidate genes from *C. bowringi* clustered with homologous genes from other Coleoptera species such as *Tribolium castaneum* (Figure S1). The functional protein domains of *C. bowringi* candidate genes were similar to those of other species such as *Homo sapiens* (Figure S2), indicating that these candidate genes were functionally conserved.

To determine whether the MAPK signaling pathway is involved in *C. bowringi* female reproduction, RNAi knockdown of these candidate genes was performed on female pupae using dsRNA microinjection. RNAi efficiency was then determined in the ovaries after 4-day PE, with results indicating that transcript levels were reduced by 55.7%–97.4% (Figure S3). According to ovarian development grades (Figure 1B), RNAi of four genes inhibited ovarian development i.e., *MEK*, *ERK*, and *KSR2* from ERK signaling pathway, and *P38* from P38 signaling pathway (Figure 1C). These findings indicate that ERK and P38 signaling pathways are important in *C. bowringi* ovarian development.

### 3.2. Effects of Knocking down Key Genes in Ovarian Development

**Figure 2:**
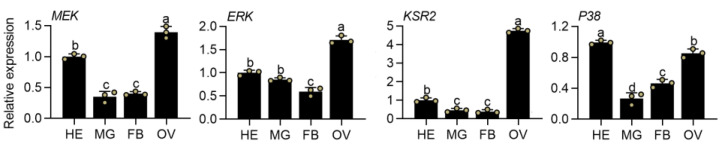
The expression patterns of *MEK*, *ERK*, *KSR2*, and *P38* in female *C. bowringi*. At 4-day PE, qRT-PCR was used to detect relative expression, and the levels of expression in the midguts (MG), fat bodies (FB), and ovaries (OV) were compared to those in the heads (HE). All data in the figures were presented as mean ± SD. The different letters above the bars denoted statistically significant differences determined by one-way ANOVA with Turkey’s LSD test (α = 0.05).

The qRT-PCR was used to detect the expression levels of key genes in heads, midguts, fat bodies, and ovaries at 4 day-PE to investigate the functions of key genes. The results revealed that the four genes were expressed in all tissues. The *MEK*, *ERK*, and *KSR2* mRNA abundance were significantly higher in the ovary compared to other tissues, while *P38* expression in the head was slightly higher than in the ovary (Figure 2). As a result, except for *KSR2*, the expression levels of other key genes showed no tissue specificity, suggesting that these genes play essential roles in multiple tissues, which may coordinately affect ovarian development.

**Figure 3:**
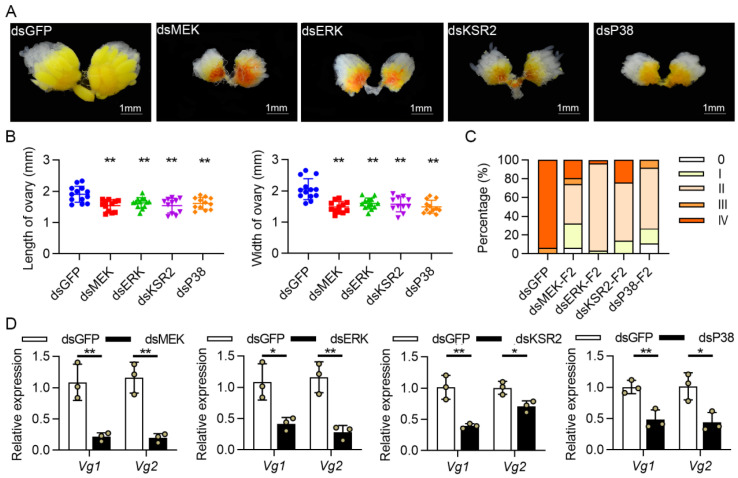
The effects of knocking down *MEK*, *ERK*, *KSR2*, and *P38* on female *C. bowringi* ovarian development. (**A**) Representative ovarian samples after dsRNA treatments. (**B**) Ovary sizes as a result of key gene silencing (*n* = 11–13). (**C**) Ovarian development grades after treatment with second non-overlapping dsRNA fragments of key genes (*n* = 18–32). (**D**) *Vg1* and *Vg2* expression levels were detected in the fat bodies. All data were collected at 4 days PE and presented as mean ± SD, with a significance test performed between the control and treatments using an independent *t*-test, * *p* < 0.05, ** *p* < 0.01.

The phenotypes of ovaries and relative expression levels of *Vg1* and *Vg2* were analyzed to further demonstrate the role of key genes in ovary development. Compared to dsGFP control, silencing key genes resulted in non-mature eggs, less vitellogenin deposition (Figure 3A), and smaller ovaries (Figure 3B). The *Vg1* and *Vg2* expression levels were significantly reduced in fat bodies following 4-day PE (Figure 3D). These findings suggested that these genes may regulate vitellogenin synthesis and play an important role in the ovarian development. To validate the RNAi results, a second fragment dsRNA targeting each key gene was designed in a non-overlapping region, and an independent RNAi experiment was carried out in the same conditions. The mRNA abundance of key genes decreased by 92.8%–96.2%, and the phenotypes of the ovaries were consistent with those of the first fragment dsRNA injection (Figure 3C and Figure S4).

### 3.3. Survivorship and Fecundity of Silencing Key Genes in Females

**Figure 4:**
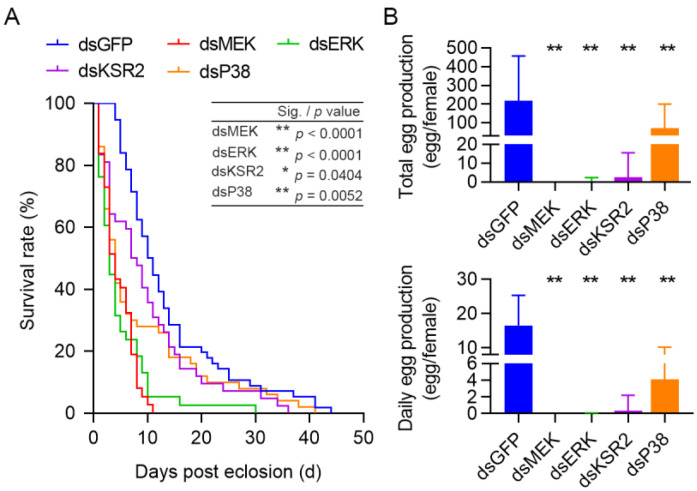
The survival rates and fecundity of silencing *MEK*, *ERK*, *KSR2*, and *P38* in female *C. bowringi*. (**A**) Survival curves of adult female beetles resulting from key gene silencing were generated using the KM survival estimate (*n* = 37–56). The log-rank test was used to compare the significance of knocking down each key gene to the dsGFP control. (**B**) Total and daily egg production per female after dsRNA injection was measured throughout the adult stage of the cabbage beetles (*n* = 17–56). All data were recorded daily and presented as mean ± SD. The significance of key gene treatments was determined using independent *t*-tests in comparison to the dsGFP control, * *p* < 0.05, ** *p* < 0.01.

Then, we investigated whether inhibiting key genes would affect the survival and fecundity of female beetles. The RNAi knockdown of *MEK* resulted in an adverse and significant decline in survival rates compared to the dsGFP control (*p* < 0.0001), resulting in the death of all beetles at 10 days PE. Knocking down *ERK* significantly increased mortality (*p* < 0.0001), whereas silencing of *KSR2* (*p* = 0.0404) and P38 (*p* = 0.0052) resulted in a modest but significant decline in adult longevity (Figure 4A).

Additional research was conducted to determine whether these key genes influence female fecundity, with total and daily egg production per female being recorded. Females with RNAi of *MEK*, *ERK*, and *KSR2* produced almost no eggs, and inhibition of P38 expression resulted in a 67.6% and 75.2% reduction in total and daily egg laying, respectively (Figure 4B), which cemented the hypothesis that *MEK*, *ERK*, *KSR2*, and *P38* were found to be critical for female *C. bowringi* survival and egg production.

### 3.4. Expression of JH, 20E and GSC Relative Genes after Knocking down Key Genes

We used qRT-PCR to detect the expression levels of JH, 20E signaling, and GSC marker genes to further understand the possible underlying mechanism of ERK and P38 pathway regulating female reproduction. The juvenile hormone acid methyltransferase 1 (JHAMT1) and Shade are critical enzymes that catalyze the production of JH and 20E, whereas Krüppel homolog 1 (Kr-h1) and E74 are key factors that respond to JH and 20E signaling [26,37,38,39]. Because JH was produced in the corpora allata (CA) and 20E was primarily produced in female adult follicle cells [47,48], we detected the expression levels of relative genes in the heads and ovaries, respectively.

**Figure 5:**
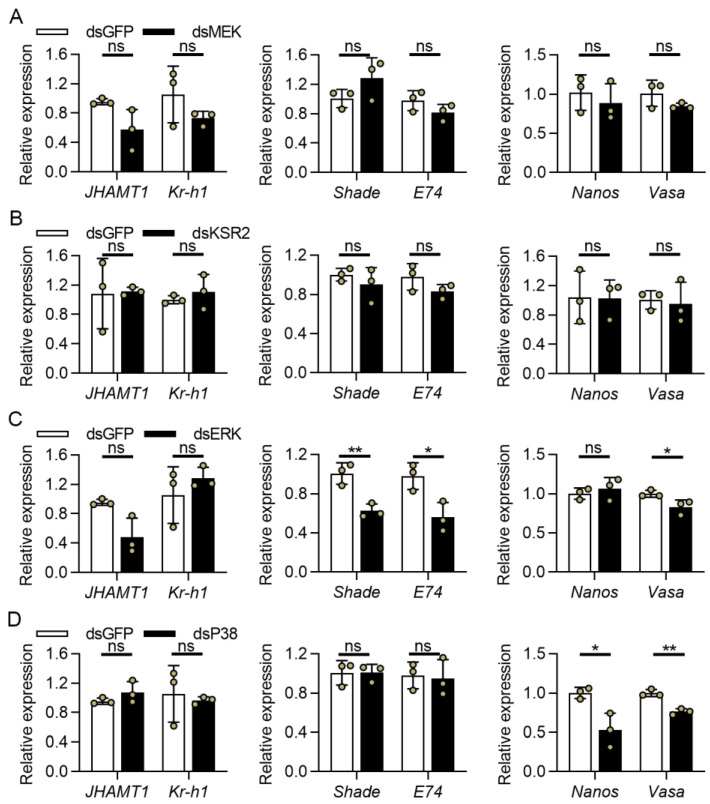
The relative expression levels of JH, 20E, and GSC marker genes in female *C. bowringi*. After dsMEK (**A**), dsKSR2 (**B**), dsERK (**C**) and dsP38 (**D**) injection, the expression levels of *JHAMT1* and *Kr-h1* were determined in the heads, and the transcript levels of *Shade*, *E74*, *Nanos*, and *Vasa* were detected in the ovaries. All data were collected at 4 day-PE and were presented as mean ± SD, ns, no significant difference, * *p* < 0.05, ** *p* < 0.01 (independent *t*-tests).

There was no significant difference in *JHAMT1* and *Kr-h1* transcript levels between the dsGFP control and the dsMEK, dsKSR2, dsERK and dsP38 treatments (Figure 5), implying that ERK and P38 pathways are not involved in JH biosynthesis. RNAi knockdown of *MEK* and *KSR2* did not induce significant difference in expression levels of *Shade*, *E74*, *Nanos* and *Vasa* (Figure 5A,B). The *ERK* inhibition resulted in a significant decrease in the expression levels of *Shade*, *E74*, and *Vasa* (Figure 5C), whereas silencing *P38* only resulted in a significant decrease in mRNA abundance of *Nanos* and *Vasa* (Figure 5D). These findings suggested that both ERK and P38 pathways could regulate GSC development and differentiation, but only the ERK signaling pathway was involved in 20E signaling. In terms of JH signaling, none of these genes appear to be involved.

## 4. Discussion

In vertebrates, the role of the MAPK signaling pathway in reproductive regulation has been explained. The ERK signaling pathway, for example, regulates critical physiological events in reproduction such as oocyte meiosis, follicle growth, and ovulation [49,50,51]. However, research on the role of the MAPK signaling pathway in female insect reproductive regulation is limited. This study comprehensively and systematically identified 14 candidate genes from the ERK, JNK, and P38 pathways in *C. bowringi*. We then performed RNAi-mediated knockdown of these genes on *C. bowringi* to identify if the MAPK signaling pathway is associated with reproduction in female insects. The current study found that only knocking down *MEK*, *ERK*, *KSR2*, and *P38* induced inactive ovaries, indicating that ERK and P38 pathways are required for ovarian development in the cabbage beetle. In contrast to recent findings that the JNK signaling pathway regulated vitellogenin (Vg) synthesis in the swimming crab *Portunus trituberculatus* [52], the knocking down of *RAC1*, *TAK1*, *MKK7*, and *JNK* did not result in any defects in yolk deposition or ovarian development in *C. bowringi*, implying that the JNK signaling pathway is not required for female insect reproduction. To the best of our knowledge, this is the first time that the function of the MAPK signaling pathway in insect ovarian development is systematically demonstrated by gene function loss.

Previous research on insects suggested that ERK signaling pathway was involved in meiotic metaphase II arrest [53], vitellogenesis [34] and eggs fertilization [54]. However, most of these studies only looked at the role of ERK signaling pathway at the cellular level, leaving it unclear whether it affects ovarian development and fecundity. Our study discovered that suppressing the transcript levels of MEK, ERK, and KSR2, MAPKK-MAPK members and ERK pathway scaffold protein [55,56,57] induced a significant halt in ovarian development as well as a complete inhibition of fecundity. Recent research has revealed that inhibiting *ERK* causes low ovariole number and fecundity in the green lacewing *Chrysopa pallens* [34], consistent with our findings. Furthermore, the function of the P38 signaling pathway during previtellogenesis has been confirmed using a *Drosophila* loss-of-function P38 MAPKK homolog mutant [58]. However, no research has been conducted to support the direct role of P38 in female insect reproduction. Our findings showed that *P38* deficiency results in immature ovaries and low egg production, implying that P38 signaling pathway is critical for female reproduction in insects. Furthermore, targeting ERK and P38 cascades reduced longevity, most likely due to inhibition of cell proliferation and reactive oxygen species signaling [59,60]. These findings suggested that RNAi-mediated control of various insect pests could have common targets. However, because these genes are evolutionarily conserved, further research should be conducted to improve target specificity and prevent apparent harm to non-target beneficial insects and other environmental organisms.

The *MEK*, *ERK*, *KSR2*, and *P38* tissue expression patterns revealed that their expression levels were higher in comparison to other tissues, indicating that ERK and P38 pathways are important for ovarian development in cabbage beetles. We detected the expression levels of critically biosynthetic and inducible JH and 20E signaling genes to further investigate the possible mechanism of these four key genes regulating female reproduction. The JH and 20E are important regulators in female insects during previtellogenesis and vitellogenesis [25], which has been proven in *C. bowringi* [37,38,39,45]. The current study found that the expression of 20E biosynthetic gene Shade and the 20E response gene E74 were significantly suppressed only after dsERK injection, implying that the ERK signaling pathway regulates 20E biosynthesis and thus influences insect female reproduction. Previous research has shown that the ERK signaling pathway is involved in 20E biosynthesis in the prothoracic gland of *D. melanogaster* [61,62], *Bombyx mori* [63], and *Leptinotarsa decemlineata* larvae [64]. A recent study revealed that *MEKK4* and *MAP2K6* participated in 20E synthesis in the female adults of *B. dorsalis*, suggesting that the MAPK signaling pathway is also involved in 20E synthesis in the ovaries [20]. However, it is unclear whether the ERK signaling pathway regulates 20E synthesis in the ovaries. Our findings suggested that the ERK signaling pathway was involved in the biosynthesis of 20E in female adults. Following that, the expression levels of key genes involved in JH biosynthesis and response were determined and the results showed no significant difference between the dsGFP control and RNAi knockdown of *ERK* and *P38*. Previous research concluded that JH activated ERK and P38 [33,65], while we proposed that ERK and P38 signaling pathways are possibly downstream of JH signaling. This study unveiled the expression of marker genes such as *Nanos* and *Vasa*, which are important in GSC development and differentiation. *Nanos* and *Vasa* were highly conserved in vertebrates and invertebrates. They were discovered in *D. melanogaster* and both responsible for primordial germ cell (PGC) determination, migration, GSC differentiation, and development [23,66,67]. The results showed that silencing *ERK* resulted in a slight decrease in *Vasa* expression, whereas silencing *P38* resulted in a decrease in *Nanos* and *Vasa* expression. In the year 1999, scientists proposed that P38 was downstream of decapentaplegic signaling and appears to be involved in the localization and translation of *Nanos* and *Vasa* [68,69]. Our findings suggest that the P38 signaling pathway may regulate GSC development and differentiation, whereas the ERK signaling pathway may only regulate PGC determination. Interestingly, our results also showed that silencing *MEK* and *KSR2* had no effect on JH and 20E biosynthesis or GSC development and differentiation. This finding implied that there might be functional compensation in the ERK signaling pathway, and *MEK* and *KSR2* probably play important roles in female reproduction independent of the ERK signaling pathway. Therefore, a further study that investigating how these two genes regulate female reproduction in *C. bowringi* would be interesting.

## 5. Conclusions

In conclusion, the current study systematically identified the genes of MAPK signaling pathways and examined the functional differentiation of ERK, JNK, and P38 pathways in cabbage beetle female reproduction at the transcriptional level. According to our findings, the ERK signaling pathway may influence reproduction by regulating 20E biosynthesis, whereas the P38 signaling pathway most likely regulates GSC development and differentiation. This study unveiled the importance of the MAPK signaling pathway in female insect reproduction and advanced our understanding of the molecular mechanisms of insect reproductive regulation.

## Data Availability

The data presented in this study are available within this article.

## Supplementary Materials

The following supporting information can be downloaded at: https://www.mdpi.com/article/10.3390/cells11101602/s1, Figure S1: The phylogenetic trees of 14 candidate genes from MAPK signaling pathway in *C. bowringi*; Figure S2: The protein domains of candidate genes from MAPK signaling pathway in *C. bowringi*; Figure S3: The efficacy of RNAi knockdown of candidate genes; Figure S4: The effect of knocking out another non-overlapping region of key genes in *C. bowringi* ovarian development.; Table S1: The blastp analysis of candidate genes for RNAi; Table S2: Primers for qRT-PCR; Table S3: Primers for RNAi.
