# Supplementary material for: MAPK Signaling Pathway Is Essential for Female Reproductive Regulation in the Cabbage Beetle, Colaphellus bowringi
Source: Cells. 2022 May 10;11(10):1602. doi: 10.3390/cells11101602 (PMC9140119; doi:10.3390/cells11101602)
Supplement: Supplementary file 1 [file cells-11-01602-s001.zip › cells-1664948-Supplementary.pdf]

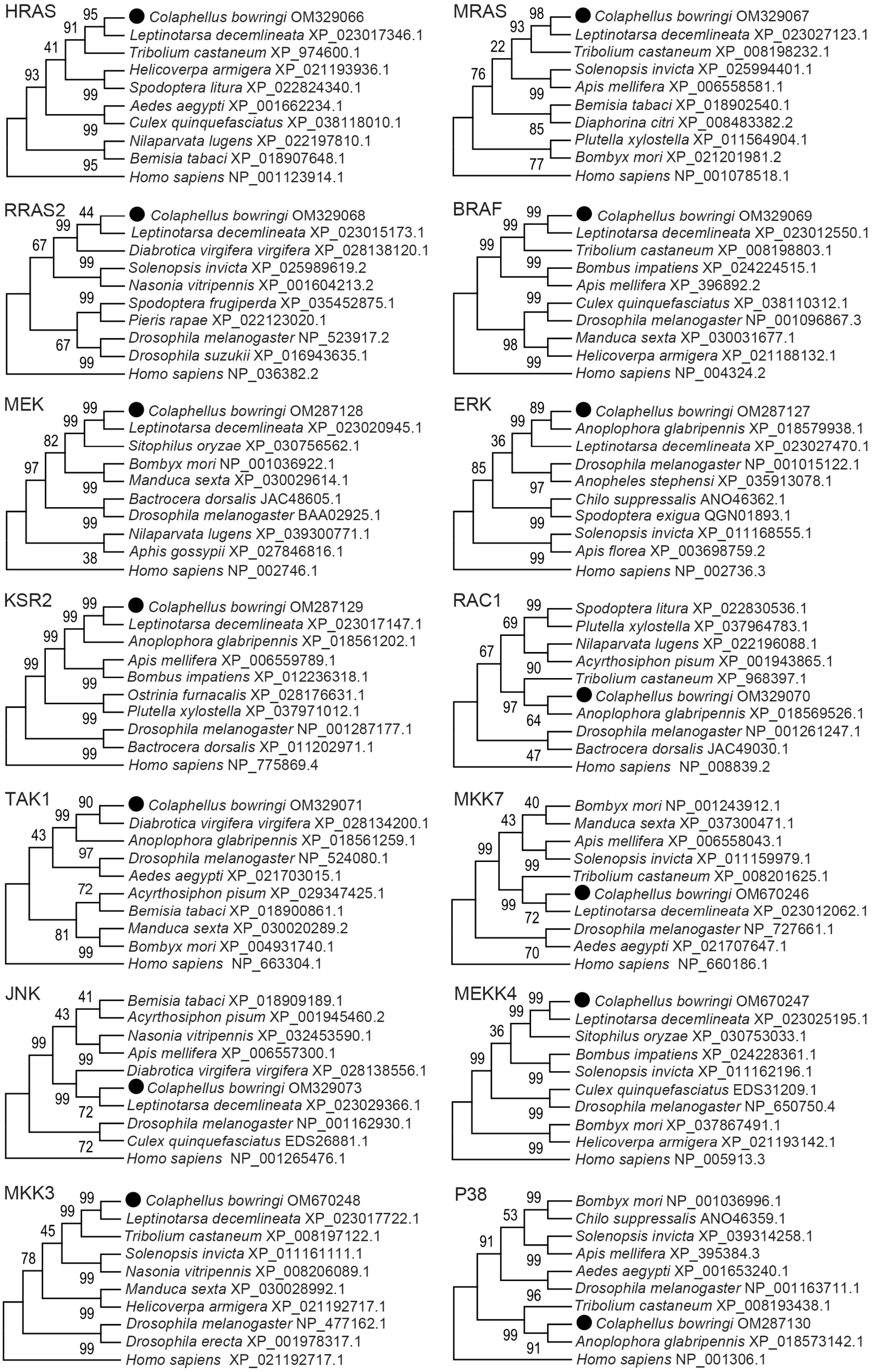
HRAS
Colaphellus bowringi OM329066
Leptinotarsa decemlineata XP_023017346.1
Tribolium castaneum XP_974600.1
Helicoverpa armigera XP_021193936.1
Spodoptera litura XP_022824340.1
Aedes aegypti XP_001662234.1
Culex quinquefasciatus XP_038118010.1
Nilaparvata lugens XP_022197810.1
Bemisia tabaci XP_018907648.1
Homo sapiens NP_001123914.1
MRAS
Colaphellus bowringi OM329067
Leptinotarsa decemlineata XP_023027123.1
Tribolium castaneum XP_008198232.1
Solenopsis invicta XP_025994401.1
Apis mellifera XP_006558581.1
Bemisia tabaci XP_018902540.1
Diaphorina citri XP_008483382.2
Plutella xylostella XP_011564904.1
Bombyx mori XP_021201981.2
Homo sapiens NP_001078518.1
RRAS2
Colaphellus bowringi OM329068
Leptinotarsa decemlineata XP_023015173.1
Diabrotica virgifera virgifera XP_028138120.1
Solenopsis invicta XP_025989619.2
Nasonia vitripennis XP_001604213.2
Spodoptera frugiperda XP_035452875.1
Pieris rapae XP_022123020.1
Drosophila melanogaster NP_523917.2
Drosophila suzukii XP_016943635.1
Homo sapiens NP_036382.2
BRAF
Colaphellus bowringi OM329069
Leptinotarsa decemlineata XP_023012550.1
Tribolium castaneum XP_008198803.1
Bombus impatiens XP_024224515.1
Apis mellifera XP_396892.2
Culex quinquefasciatus XP_038110312.1
Drosophila melanogaster NP_001096867.3
Manduca sexta XP_030031677.1
Helicoverpa armigera XP_021188132.1
Homo sapiens NP_004324.2
MEK
Colaphellus bowringi OM287128
Leptinotarsa decemlineata XP_023020945.1
Sitophilus oryzae XP_030756562.1
Bombyx mori NP_001036922.1
Manduca sexta XP_030029614.1
Bactrocera dorsalis JAC48605.1
Drosophila melanogaster BAA02925.1
Nilaparvata lugens XP_039300771.1
Aphis gossypii XP_027846816.1
Homo sapiens NP_002746.1
ERK
Colaphellus bowringi OM287127
Anoplophora glabripennis XP_018579938.1
Leptinotarsa decemlineata XP_023027470.1
Drosophila melanogaster NP_001015122.1
Anopheles stephensi XP_035913078.1
Chilo suppressalis ANO46362.1
Spodoptera exigua QGN01893.1
Solenopsis invicta XP_011168555.1
Apis florea XP_003698759.2
Homo sapiens NP_002736.3
KSR2
Colaphellus bowringi OM287129
Leptinotarsa decemlineata XP_023017147.1
Anoplophora glabripennis XP_018561202.1
Apis mellifera XP_006559789.1
Bombus impatiens XP_012236318.1
Ostrinia furnacalis XP_028176631.1
Plutella xylostella XP_037971012.1
Drosophila melanogaster NP_001287177.1
Bactrocera dorsalis XP_011202971.1
Homo sapiens NP_775869.4
RAC1
Spodoptera litura XP_022830536.1
Plutella xylostella XP_037964783.1
Nilaparvata lugens XP_022196088.1
Acyrthosiphon pisum XP_001943865.1
Tribolium castaneum XP_968397.1
Colaphellus bowringi OM329070
Anoplophora glabripennis XP_018569526.1
Drosophila melanogaster NP_001261247.1
Bactrocera dorsalis JAC49030.1
Homo sapiens NP_008839.2
TAK1
Colaphellus bowringi OM329071
Diabrotica virgifera virgifera XP_028134200.1
Anoplophora glabripennis XP_018561259.1
Drosophila melanogaster NP_524080.1
Aedes aegypti XP_021703015.1
Acyrthosiphon pisum XP_029347425.1
Bemisia tabaci XP_018900861.1
Manduca sexta XP_030020289.2
Bombyx mori XP_004931740.1
Homo sapiens NP_663304.1
MKK7
Bombyx mori NP_001243912.1
Manduca sexta XP_037300471.1
Apis mellifera XP_006558043.1
Solenopsis invicta XP_011159979.1
Tribolium castaneum XP_008201625.1
Colaphellus bowringi OM670246
Leptinotarsa decemlineata XP_023012062.1
Drosophila melanogaster NP_727661.1
Aedes aegypti XP_021707647.1
Homo sapiens NP_660186.1
JNK
Bemisia tabaci XP_018909189.1
Acyrthosiphon pisum XP_001945460.2
Nasonia vitripennis XP_032453590.1
Apis mellifera XP_006557300.1
Diabrotica virgifera virgifera XP_028138556.1
Colaphellus bowringi OM329073
Leptinotarsa decemlineata XP_023029366.1
Drosophila melanogaster NP_001162930.1
Culex quinquefasciatus EDS26881.1
Homo sapiens NP_001265476.1
MEKK4
Colaphellus bowringi OM670247
Leptinotarsa decemlineata XP_023025195.1
Sitophilus oryzae XP_030753033.1
Bombus impatiens XP_024228361.1
Solenopsis invicta XP_011162196.1
Culex quinquefasciatus EDS31209.1
Drosophila melanogaster NP_650750.4
Bombyx mori XP_037867491.1
Helicoverpa armigera XP_021193142.1
Homo sapiens NP_005913.3
MKK3
Colaphellus bowringi OM670248
Leptinotarsa decemlineata XP_023017722.1
Tribolium castaneum XP_008197122.1
Solenopsis invicta XP_011161111.1
Nasonia vitripennis XP_008206089.1
Manduca sexta XP_030028992.1
Helicoverpa armigera XP_021192717.1
Drosophila melanogaster NP_477162.1
Drosophila erecta XP_001978317.1
Homo sapiens XP_021192717.1
P38
Bombyx mori NP_001036996.1
Chilo suppressalis ANO46359.1
Solenopsis invicta XP_039314258.1
Apis mellifera XP_395384.3
Aedes aegypti XP_001653240.1
Drosophila melanogaster NP_001163711.1
Tribolium castaneum XP_008193438.1
Colaphellus bowringi OM287130
Anoplophora glabripennis XP_018573142.1
Homo sapiens NP_001306.1

Figure S1. The phylogenetic trees of 14 candidate genes from MAPK signaling pathway in *C. bowringi*. ExPASy translate tool was used to determine the protein sequences of candidate genes. The 14 rooted phylogenetic trees were built in MEGA 6 using the neighbor-joining method. Protein sequences used in phylogenetic analyses came from various insect orders, including Coleoptera, Diptera, Lepidoptera, Hemiptera, and Hymenotera.

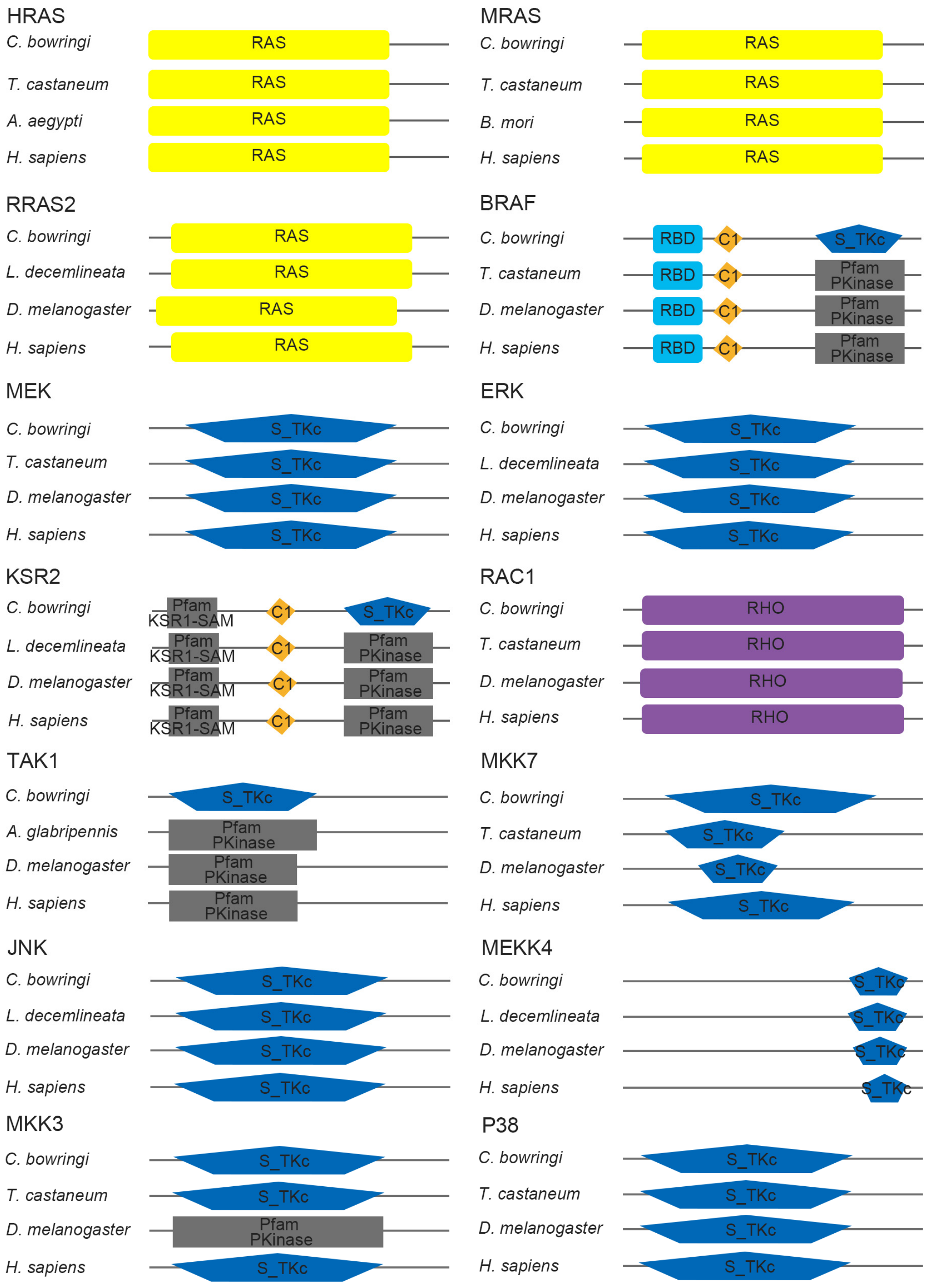

Figure S2. The protein domains of candidate genes from MAPK signaling pathway in *C. bowringi*. The ExPASy translate tool was used to deduce protein sequences of candidate genes, and SMART tool was used to predict protein domains. The protein sequences (the accession numbers were shown in Fig. S1) used for protein domains prediction came from a

variety of species, including *Colaphellus bowringi*, *Tribolium castaneum*, *Leptinotarsa decemlineata*, *Anoplophora glabripennis*, *Bombyx mori*, *Aedes aegypti*, *Drosophila melanogaster*, and *Homo sapiens*.

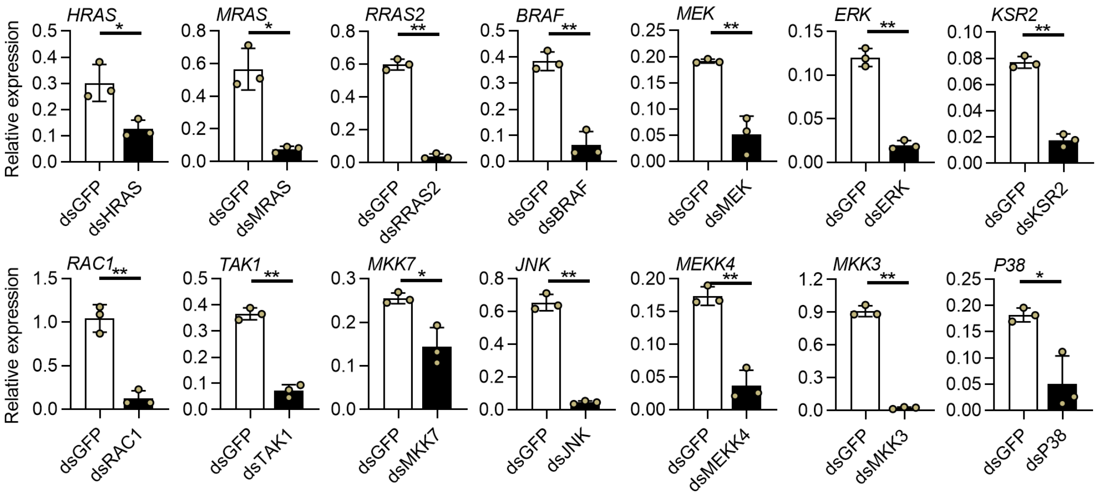

Figure S3. The efficacy of RNAi knockdown of candidate genes. After injecting dsRNAs into female pupae of *C. bowringi*, the expression levels of 14 candidate genes were detected in the ovaries. All data were collected at 4-day PE and presented as mean ± SD, and the significance test was performed with an independent *t*-test, * $P < 0.05$, ** $P < 0.01$.

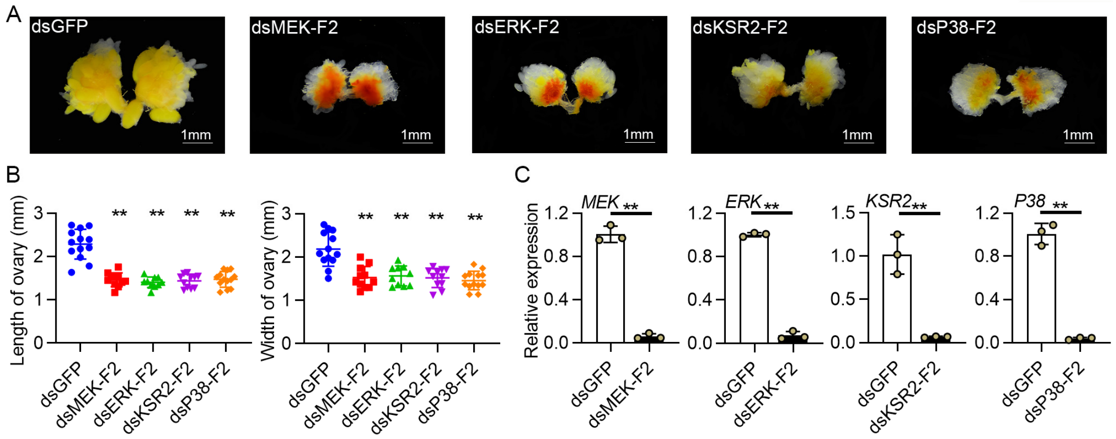

Figure S4. The effect of knocking down another non-overlapping region of key genes in *C. bowringi* ovarian development. (A) Representative ovarian samples following 2 μg dsRNA microinjection into female pupae. (B) The size of ovaries as a result of silencing key genes n = 10–13. (C) The expression levels of key genes in the ovaries were measured. All data were collected at 4-day PE and presented as mean ± SD, with the significance test using independent *t*-test, * P < 0.05, ** P < 0.01.

Table S1. The blastp analysis of candidate genes for RNAi

Gene (Abbreviation)	Gene ID	Closest species	Genbank number	E value
GTPase HRas (*HRAS*)	CL223.Contig3_All	*Leptinotarsa decemlineata*	XP_023017346.1	7E-122
Ras-related protein M-Ras (*MRAS*)	Unigene2066_All	*Anoplophora glabripennis*	XP_018563656.1	2E-141
Ras-related protein R-Ras2 (*RRAS2*)	Unigene1450_All	*Leptinotarsa decemlineata*	XP_023015173.1	1E-146
B-Raf proto-oncogene serine/threonine-protein kinase (*BRAF*)	CL3151.Contig3_All	*Leptinotarsa decemlineata*	XP_023012550.1	0
Mitogen-activated protein kinase kinase 1(*MEK*)	Unigene14333_All	*Anoplophora glabripennis*	XP_018576061.1	0
Mitogen-activated protein kinase 1 (*ERK*)	CL5074.Contig3_All	*Leptinotarsa decemlineata*	XP_023027470.1	0
Kinase suppressor of Ras 2 (*KSR2*)	Unigene549_All	*Leptinotarsa decemlineata*	XP_023017147.1	0
Ras-related C3 botulinum toxin substrate 1 (*RAC1*)	CL2204.Contig2_All	*Anoplophora glabripennis*	XP_018569526.1	7E-139
Mitogen-activated protein kinase kinase kinase 7 (*TAK1*)	CL5016.Contig2_All	*Leptinotarsa decemlineata*	XP_023017563.1	0
Mitogen-activated protein kinase kinase 7 (*MKK7*)	CL3117.Contig2_All	*Leptinotarsa decemlineata*	ALE20541.1	0
C-Jun N-terminal kinase (*JNK*)	CL1663.Contig3_All	*Leptinotarsa decemlineata*	XP_008197492	0
Mitogen-activated protein kinase kinase kinase 4 (MEKK4)	Unigene5981_All	*Leptinotarsa decemlineata*	XP_023025197.1	0
Mitogen-activated protein kinase kinase 3 (*MKK3*)	CL3731.Contig2_All	*Leptinotarsa decemlineata*	XP_023017722.1	0
P38 MAP kinase (*P38*)	Unigene12015_All	*Anoplophora glabripennis*	XP_018573142.1	0

Table S2. Primers for qRT-PCR

Genes	Forward primers (5'-3')	Reverse primers (5'-3')	PCR efficiency (%)	Standard curve R^2
RPL19	gtaatgcgatgcggcaagaa	gagtgcaccgctacaggttt	102.2	0.998
Actin1	tcaagcggtgttagctctgg	tatcgatcacgatgccggtg	100.2	0.996
HRAS	ggagacatcagccaagac	agcacctcaacttgaatgg	100.4	0.999
MRAS	gctcacaaattgggcattc	ggattattctcaccacctcat	107.6	0.999
RRAS2	agagtagtttggcaagaaga	agcattatcgacgttcattc	112.2	1.000
BRAF	agcaattccaccaacctac	gcaactgaccagacaaca	99.2	0.999
MEK	ttgaagggtttgagctatct	tctctggcgacatataactt	104.2	0.999
ERK	cgatactgagttggatgatt	tggttctatgtgatgcctt	101.3	0.998
KSR2	ggactcttcagtgtgacaa	gtagacttctcataacttctgg	96.2	0.997
RAC1	ccaatcacatatccacagg	gcatcacaggacatagtac	111.8	0.995
TAK1	accaacacaatgtcagagaa	agtgctgttccatattcctt	114.2	0.998
MKK7	accgaatctgaagtatggat	taaggcttctactgttgcta	106.2	0.996
JNK	cattgtatgtgctgctgtc	ggagaaggcgttcagtaa	96.9	0.998
MEKK4	ggaatgtaagcaaggttgtt	tatagcgtctgtaaggtgag	99.3	0.997
MKK3	cctcctcggttaccttca	tggctgtagttcggtcta	109.6	0.998
P38	cattatcagcagttggttca	agcagtttcagttccctata	96.9	0.999
Vg1	ggaatgcgcgtgagattgac	cgaagcagttttgtggggtg	100.2	0.996
Vg2	tggaccgattcacaaccctg	tcgaagatgaggaatgcggg	105.4	0.997
JHAMT1	gggttttcagtgtagattctgga	cgaaaatcagtttgtccctctgt	91.5	0.998
Kr-h1	caactcaaagtgcacacccg	ccgtaatgcgcaacctgatg	102.0	0.998
Shade	actgacgagcccaaaaacca	acgacgtttcgagacccaat	106.0	0.999
E74	agggattcgggcactgaaag	tgcctcgaaagtagctggtg	100.7	0.999
Nanos	ctttccaacggtgtcaaa	gaaacagaaacaactaagcc	108.5	0.994
Vasa	tgtatcaccaacacgagaat	gacagccactcaacacat	112.0	0.996

Table S3. Primers for RNAi

Genes	dsRNA region	Forward primers (5'-3')	Reverse primers (5'-3')
GFP	region 1	gcgtaatacgactcactataggtggtcccaattctcgtggaac	gcgtaatacgactcactataggcttgaagttgaccttgatgcc
HRAS	region 1	gcgtaatacgactcactataggcagcattgaccattcagttg	gcgtaatacgactcactataggtcatatccacagcccagttt
MRAS	region 1	gcgtaatacgactcactataggtagtcgtagttggagatgga	gcgtaatacgactcactataggtcggataagtgtctctgtct
RRAS2	region 1	gcgtaatacgactcactataggattagttatcgtgggtggag	gcgtaatacgactcactataggaaccttattgccgaccataa
BRAF	region 1	gcgtaatacgactcactataggtggaacaactgagtaatgga	gcgtaatacgactcactataggttggtgacataaggaaggaa
MEK	region 1	gcgtaatacgactcactataggccaactgtaactccgacttc	gcgtaatacgactcactataggaatcaagagaaccagcatcc
	region 2	gcgtaatacgactcactataggctcagtccagagtgacatat	gcgtaatacgactcactatagggcctgctatatccacatctt
ERK	region 1	gcgtaatacgactcactataggacgatcacacagggttcctc	gcgtaatacgactcactataggctcatcagccggatcgtaat
	region 2	gcgtaatacgactcactatagggcttacgatactgtgacgaa	gcgtaatacgactcactataggtgagaagtagattgcttggt
KSR2	region 1	gcgtaatacgactcactataggaacctcacgaagttgaattg	gcgtaatacgactcactataggataggatgagatggactacg
	region 2	gcgtaatacgactcactataggatgatgtcgtcttgtggtta	gcgtaatacgactcactataggatgactgaatgtggatgtga
RAC1	region 1	gcgtaatacgactcactatagggtctcctgataagctacact	gcgtaatacgactcactataggaatagtcgctctatcgtctc
TAK1	region 1	gcgtaatacgactcactataggaggtgttcacatcatctcat	gcgtaatacgactcactataggttcctcaacttcggtgtaat
MKK7	region 1	gcgtaatacgactcactataggtgcttatatggctccagaac	gcgtaatacgactcactataggcatccgattgttgaagaagt
JNK	region 1	gcgtaatacgactcactataggccgattgcacattgaagata	gcgtaatacgactcactataggccaccgatattctactctgt
MEKK4	region 1	gcgtaatacgactcactataggacttgcagttcggaagagga	gcgtaatacgactcactatagggcaaacttgctacccaccat
MKK3	region 1	gcgtaatacgactcactataggccagtgggacaataatggct	gcgtaatacgactcactataggggttgccttggggatctatt
P38	region 1	gcgtaatacgactcactataggttgggtgtatcatggcagaa	gcgtaatacgactcactataggtcatcgttgggatcagcata
	region 2	gcgtaatacgactcactataggccgagacacgataatagtca	gcgtaatacgactcactataggaagattcaactggtggatgt

Note: T7 promoter sequence is "gcgtaatacgactcactatagg".
